# Opposing Patterns of Seasonal Change in Functional and Phylogenetic Diversity of Tadpole Assemblages

**DOI:** 10.1371/journal.pone.0151744

**Published:** 2016-03-25

**Authors:** Axel Strauß, François Guilhaumon, Roger Daniel Randrianiaina, Katharina C. Wollenberg Valero, Miguel Vences, Julian Glos

**Affiliations:** 1 Zoologisches Institut, Technische Universität Braunschweig, Mendelssohnstr. 4, 38106, Braunschweig, Germany; 2 Institut für Genetik, Ludwig-Maximilians-Universität München, Großhaderner Str. 2–4, 82152 Martinsried, München, Germany; 3 IRD, MARBEC, Université de Montpellier, Montpellier, France; 4 Département de Biologie Animale, Université d’Antananarivo, Antananarivo, 101, Madagascar; 5 College of Science, Engineering and Mathematics, Department of Natural Sciences, Bethune-Cookman University, 640 Dr. Mary McLeod Bethune Blvd., Daytona Beach, Florida, 32114, United States of America; 6 Zoologisches Institut, Universität Hamburg, Martin-Luther-King Platz 3, 20146, Hamburg, Germany; University of Sao Paulo, BRAZIL

## Abstract

Assemblages that are exposed to recurring temporal environmental changes can show changes in their ecological properties. These can be expressed by differences in diversity and assembly rules. Both can be identified using two measures of diversity: functional (FD) and phylogenetic diversity (PD). Frog communities are understudied in this regard, especially during the tadpole life stage. We utilised tadpole assemblages from Madagascan rainforest streams to test predictions of seasonal changes on diversity and assemblage composition and on diversity measures. From the warm-wet to the cool-dry season, species richness (SR) of tadpole assemblages decreased. Also FD and PD decreased, but FD less and PD more than expected by chance. During the dry season, tadpole assemblages were characterised by functional redundancy (among assemblages—with increasing SR), high FD (compared to a null model), and low PD (phylogenetic clustering; compared to a null model). Although mutually contradictory at first glance, these results indicate competition as tadpole community assembly driving force. This is true during the limiting cool-dry season but not during the more suitable warm-wet season. We thereby show that assembly rules can strongly depend on season, that comparing FD and PD can reveal such forces, that FD and PD are not interchangeable, and that conclusions on assembly rules based on FD alone are critical.

## Introduction

The properties of species assemblages vary in time. On an ecological time scale, these variations might be anthropogenically caused, e.g., by drastic single events (such as fire), or recurring due to annual climatic changes [1–4]. Most obviously, changes in species assemblages are reflected by changes in species richness (SR). However, SR as measure of diversity is likely to miss relevant information because ecological traits and phylogenetic relatedness of species in an assemblage are not independent. Ecological traits of species (and therefore the functional diversity of assemblages, FD) can determine their ability to exploit resources, influence potential of coexistence, and may reflect the ecological impact of assemblages (i.e., ecosystem functions such as biomass production [5–7]. The degree of relatedness (summarised as the phylogenetic diversity of an assemblage, PD), is often used as alternative measure of assemblage structure and its influence on ecosystem processes [8]. The information needed to measure PD (i.e., a phylogenetic tree usually based on molecular data) is easier and less expensive to obtain, and often less ambiguous, than complex ecological data on species, and PD has been proposed as a proxy for FD [9, 10]. PD can be [11] but is not necessarily [12] a better determinant of diversity than FD. Whether PD indeed well reflects ecological functions in species assemblages has been questioned [13, 14] and exceptions have been found [11, 15, 16].

Such non-congruency between FD and PD can be caused by different factors. It is often assumed that closely related species show a high degree of morphological and ecological similarity [17]. PD covers more dimensions than the number of traits typically used to calculate FD [10]; however, it does not provide information on what these dimensions are [10]. Furthermore, PD is based on the assumption that relevant traits are conserved across phylogenies but there are cases of traits missing a phylogenetic signal (e.g., [18, 19]), or of a strong variation in phylogenetic signal of traits [20], usually due to extensive homoplasy. Since PD is easy to access and includes evolutionary history as well as unmeasured traits [21] it is still considered valuable if handled (and interpreted) with care [22] and can be a good predictor of ecosystem functioning [10].

The influence exerted by competition on FD and PD of species assemblages is usually tested by comparison of observed with randomly assembled "null assemblages"—consisting of members of the local species pool (e.g., [9, 15, 23–25]). If observed assemblages have higher FD (simply called "high FD") than the null assemblages, this is understood as an indication of competition as a relevant factor in shaping these assemblages, whereas low FD is an indicator of environmental filtering [23, 24]. The competition-relatedness hypothesis [26] transfers this interpretation to PD. However, the role of competition and environmental filtering in shaping assemblages has continuously been debated and alternative outcomes on FD and PD have been suggested [27]. Studies of PD combined with quantitative ecological traits will help interpreting relatedness in assemblages and environmental conditions shaping them.

If environmental conditions change, subsequent compositional changes of species assemblages may affect FD and PD in a different way than it would affect SR. Two different processes have been characterized by which such effects can lead to functional redundancy. *Intrinsic functional redundancy* (sensu [24]) occurs for instance if an assemblage, as a starting point, contains a high proportion of functionally similar species. In such a case, *random* decreases of species numbers will have a lower effect on FD than on SR [24]. *Extrinsic functional redundancy* instead originates by a process of *non-random* change in a species assemblage, e.g., when species disappearing from an assemblage are mostly functionally unique. These concepts can be extended to intrinsic or extrinsic phylogenetic redundancy leading to phylogenetic overdispersion or clustering regarding relatedness [28, 29]. Redundancy obviously is low in opposite situations, i.e., assemblages consist of high proportions of unique species (intrinsic), or species disappearing from the communities are mostly similar (extrinsic). Intrinsic redundancy has been observed in a range of disturbed ([19, 30] but see [23, 24]) and undisturbed [25] ecosystems across several taxa regarding species function (i.e., FD), and in urban plant assemblages [31] regarding relatedness (i.e., PD). Examples for extrinsic redundancy are observed in directly human influenced systems across several animal and plant taxa [30, 32, 33].

Tropical anuran assemblages represent an appropriate model to study seasonal changes and their impact on different measures of diversity as they are known to be remarkably rich but still can be completely assessed and taxonomically handled [34]. Seasonal changes in SR, i.e., seasonal changes in frog (reproductive) activity, have been observed for adult amphibians [35–37], and to some extent for amphibian larval assemblages [38].

In this study we focus on the world’s most species rich stream tadpole assemblages in the rainforests of Madagascar [25] to evaluate seasonal patterns of SR, FD and PD. We expect SR to be lower in the dry season than in the wet season, following the pattern observed for adults [35]. Subsequently, we analyze according seasonal changes in FD and PD, and compare these changes against null models to identify high or low FD and PD, and functional redundancy and phylogenetic clustering or overdispersion, respectively.

## Methods

### Study sites

**Fig 1:**
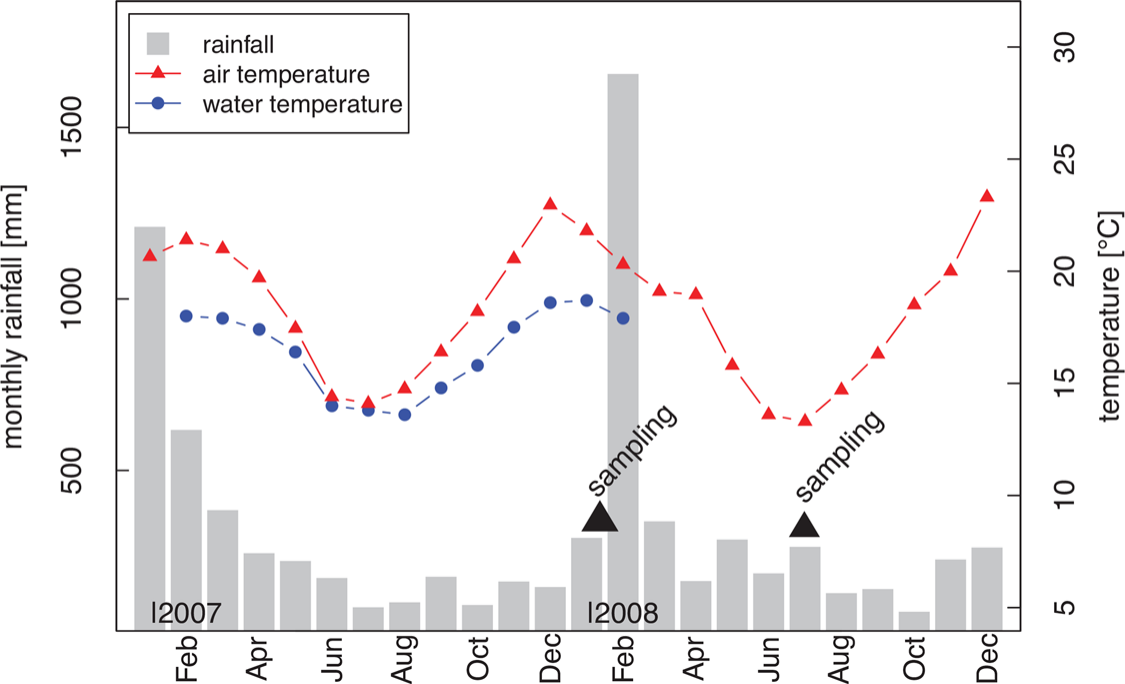
Annual changes in precipitation and temperature. Annual changes in precipitation and temperature from the RNP region from January 2007 to December 2008. The total monthly precipitation is given in grey bars. The curves represent the mean monthly temperature retrieved from daily minimum-maximum-measurements of air temperature (red triangles) and the stream water temperature (blue circles; data from Fompohonina River). Data of rainfall and air temperature were provided by ValBio research station, water temperature was retrieved from iButton temperature loggers.

We conducted fieldwork in the wet season (January and February) and the dry season (July) of 2008. Study sites were located in one of the centres of amphibian species richness in Madagascar, Ranomafana National Park (RNP; 21°16'S; 47°25'E). RNP covers over 40,000 ha of rain forest from ca. 500 m up to ca. 1.500 m a.s.l. and harbours over 100 frog species [34]. Seasonality in this area is characterised by clear differences in precipitation and temperature (Fig 1). Activity patterns of adult frogs in Ranomafana are different between seasons [35].

During the austral winter dry season, the precipitation in RNP can reach values as low as <100 mm in some months, and temperatures are distinctly cooler than in summer. The wet season in the austral summer is generally characterised by high precipitation but the maximum can vary between months (see e.g. January 2007 *vs*. January 2008 and February 2007 *vs*. February 2008; see also Wollenberg et al. [39]).

### Species sampling

We sampled tadpole assemblages by intensive capture in 30 m sections of 12 streams (in a mid-elevational area ranging from 910 m to 1.130 m a.s.l.). Sampling details are provided in Strauß et al. [40]. We kept tadpoles alive and carried them in water containers into the laboratory. They were euthanized by immersion in MS222 solution, and immediately sorted into series based on their morphology. From each series, we identified one specimen by DNA barcoding based on a fragment of the mitochondrial *16S* rRNA gene [41, 42]. All newly determined DNA sequences have been deposited in Genbank under accession numbers KF609548-KF611386. Sampling was carried out once in the wet and once in the dry season, applying the same sampling methods to the same stream sections. All analyses are based exclusively on tadpole data which are a reliable representation of the frog species actually breeding in a single stream; sightings of adults were not considered.

We tracked water temperature during the seasons by placing temperature loggers (Thermochron® iButton, Dallas Semiconductor) in the streams one year before this study. They conducted measurements every 255 min covering about one year (early 2007 to early 2008). Data on aerial temperature and rainfall were provided by the ValBio research station, Ranomafana (J. C. Razafimahaimodison).

### Ethics Statement

No experiments were conducted using living animals.

All field research, including collection of specimens using dip nets, carriage to the lab in water containers, anaesthesia, and euthanasia of specimens, were approved by the Madagascan Ministére de l’Environnement, des Eaux et des Forêts (Direction des Eaux et Forêts, DEF) under the following permits: 300/06/MINENV.EF/SG/DGEF/DPB/SCBLF/RECH dated 22 December 2006; 003/08-MEEFT/SG/DGEF/DSAP/SSE dated 4 January 2008; 004/08-MEEFT/SG/DGEF/DSAP/SSE dated 4 January 2008; and MEEFT/SG/DGEF/DSAP/SSE dated 30 January 2008. Export of specimens was approved by the DEF under permit: 052N-EA02/MG08 dated 28 February 2008. Sampling for this study was conducted in January, February, and July 2008. No species protected by the Convention of the International Trade in Endangered Species CITES were concerned by this research. Voucher specimens were euthanized using approved methods (e.g., anaesthesia with MS222, followed by overdose of the same substance and 95% ethanol fixation). These are standard methods included in the permits above that do not require approval by an ethics committee. Killings were recorded and reported to Madagascan authorities as requested in the respective authorisations (included in the permits stated above).

### Statistical analyses

**Fig 2:**
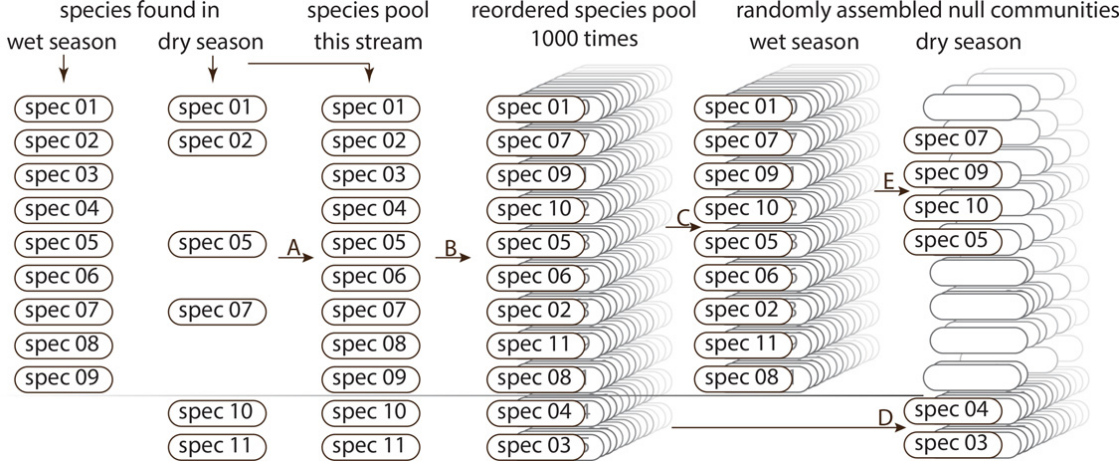
Scheme illustrating process of setting null assemblages. Scheme illustrating process of setting null assemblages based on observations in the wet and the dry season. (A)–all species found are pooled. (B)–This species pool is randomly ordered a thousand times. (C)–null assemblages for wet season are retrieved: the first nine species if there have been nine species observed in the wet season. (D)–for the null assemblages of the dry season, first the remaining (here two) species that have not been used for the respective wet season assemblage are taken and (E)–filled up by randomly chosen species from the respective wet season assemblage (here four) until the original species richness of the dry season assemblage is reached.

We assessed species richness (SR) of tadpole assemblages in dry and wet season based on molecular identification of tadpoles sampled. We first confirmed the expectation that species richness (SR) of tadpole assemblages differs between wet and dry season by paired t-test (all software details are stated and referenced at the end of this section). Before testing whether changes in FD or PD are similar to changes in SR or whether species loss and turnover depend on the ecological function of species, we developed a null model. For this null model, we remodelled random assemblages ("null assemblages") of wet and dry season. Modelling was based on observed SR of assemblages in both seasons and therefore the observed changes in SR and the observed species turnover, as well as the observed species pool available for each stream. In detail, for each stream we pooled all species found in wet and/or dry season in this stream (= species pool available). For example, if nine species were found in the wet season and six in the dry season of which two were not sampled in the wet season, the species pool consisted of 9+2 (11) species (Fig 2A). We reordered them randomly 1000 times (Fig 2B). Out of these 1000 species pools, we each picked the first x species (with x being the number of species observed for this stream in the wet season; nine in our example) and thus achieved null assemblages for the wet season (Fig 2C). To achieve null assemblages for the dry season, we first picked each the remaining y species that were not already included in wet season assemblages (with y being the number of species that were found in the dry but not in the wet season in this stream; two in our example; Fig 2D) and then restocked with randomly chosen species from the respective random wet season assemblage (four in our example; Fig 2E) to reach the final number of species for the random dry season assemblage as observed (six in our example). This was repeatedly done for each stream based on the species that we observed in the stream. For each stream and season we first calculated FD of the tadpole null assemblages in the seasons and then the change in FD from the wet to the dry season, also based on the null assemblages. To calculate the relative change of diversity from the wet season (“wet”) to the dry season (“dry”) we developed an approach using –1*(1–dry/wet) resulting in positive values indicating an increase and negative values indicating a decrease in the respective diversity measure.

FD was calculated following Petchey & Gaston [43, 44]. This method applies a three-step dendrogram based classification function in which a species trait matrix is used to calculate a pair-wise species distance matrix. This matrix is used to construct dendrograms of specific species assemblages. The total branch length needed to connect all species in the assemblage represents the respective FD. Best distance measure (Gower’s distance) and cluster method (UPGMA) were identified following Mouchet et al. [45]. Our trait matrix consisted of categorical and continuous morphological trait variables (oral disc, body shape) of ecological relevance (i.e. feeding, microhabitat choice; [46]) for all species (for a list of traits used, see [25]).

We calculated FD and the relative changes in FD for the observed assemblages, and for null assemblages (see above). We compared relative changes of observed and null assemblage FD using paired t-test. Accordingly, significant differences show that seasonal changes in FD are not random.

Non randomness can be caused by differences in redundancy patterns, or by high or low FD in one or both seasons [24]. We therefore analysed data of the wet and the dry season separately. We applied polynomial regression with observed FD as dependent and SR (linear term) and SR^2 (polynomial term) as independent variables to reveal possible patterns of functional redundancy in each of both seasons (i.e., if nonlinearity is found). To test whether the full polynomial model or a simplified (i.e., linear) model performs better we used stepwise deletion (polynomial term first) and compared the models based on Akaike’s Information Criterion (AIC, [47, 48]) until the minimum adequate models were reached. Residuals were checked using diagnostic plots. We used Moran’s I autocorrelation coefficient [49] to prove that there is no spatial autocorrelation of the study sites regarding SR, FD, and PD, and their respective changes between wet and dry season. In this process no functional redundancy is indicated if these analyses show only a linear relationship between FD and SR (i.e., if the linear term in the polynomial model happens to be significant and the polynomial term to be non significant). Functional redundancy is indicated if the slope of the relationship between FD and SR decreases with increasing SR (i.e., if the polynomial term in the model is significant), caused by a stronger overlap of ecological traits with increasing number of syntopic tadpole species. We compared observed FD data with the respective null model data using paired t-tests, separately for each season. This allowed identifying possible patterns of low or high FD in the tadpole assemblages if differences were significant. All t-test were two-sided Welch t-tests.

We conducted the same procedure to analyse patterns of phylogenetic diversity (PD) and its respective changes from wet to dry season. Our analyses are based on a time-calibrated phylogeny of mantellid species [50] as all tadpoles sampled in the streams belonged to this family [40]. We pruned from this tree all taxa not represented in our Ranomafana tadpole sampling using TreeEdit version v1.0a10. This reduced the number of species by retaining branch lengths. For a few so far undescribed species, genetic data were insufficient to include them in the tree. In these cases, other species that are known to be in the same clade (according to molecular data of Vieites et al. [34]) were used as replacement for the purpose of PD calculation. Similar to FD calculations [43, 44, 51], we extracted branch lengths from the tree for the assemblages [52] by using the function treedive included in the R package vegan. The sum of branch lengths needed to connect all species of an assemblage represents the assemblage’s PD.

All analyses were run using the statistical software R 2.15.1 [53]. Packages used for FD calculations include car R package version 2.0–18 [54], gtools R package version 2.7.0. [55], cluster R package version 1.14.2 [56] and clue R package version 0.3–45 [57, 58]. For PD calculations we used the R packages ape version 3.0–5 [59, 60] and vegan version 2.0–4 [61]. Moran’s I was also calculated using the package ape.

## Results

Summarizing data over wet and dry seasons, we found tadpoles of a total of 31 species in all twelve stream sections (= assemblages). In the wet season we found five to 15 species, in the dry season two to twelve species per assemblage. Mean SR in the dry season was about 27% lower than in the wet season (paired t-test, t = 3.44, df = 11, p = 0.006). Beside this decrease in SR (i.e., probably due to reduced reproductive activity), a species turnover from wet to dry season was observed in eight streams, i.e., an assemblage in the dry season harboured on average one or more species that were not present in the assemblage of the same stream during the wet season. However, no species was exclusively found in the dry season.

### Functional diversity

**Fig 3:**
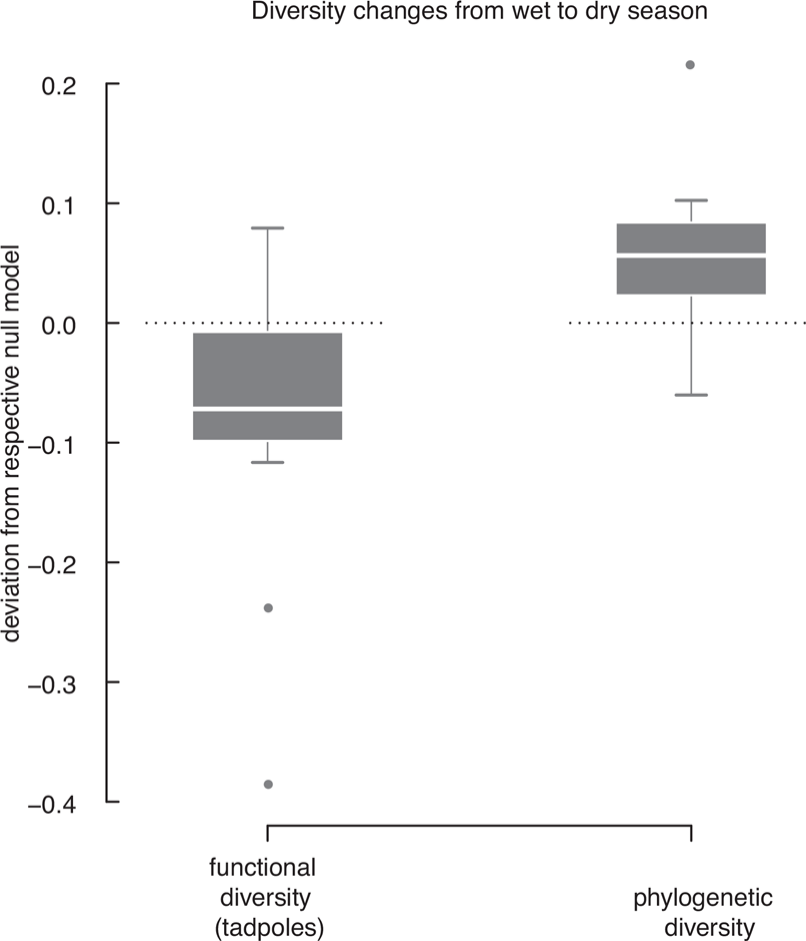
Change from the wet to the dry season: Deviation of observed loss of FD and PD from the null models. The dashed lines represent the null models for the change from the wet to the dry season, the box-whisker-plots show the observed deviations from the null models. Values below the dashed line show a smaller loss, values above the line show a higher loss of FD and PD than predicted by the null models. FD of tadpoles decreases significantly less than predicted whereas PD decreases significantly more than predicted.

**Fig 4:**
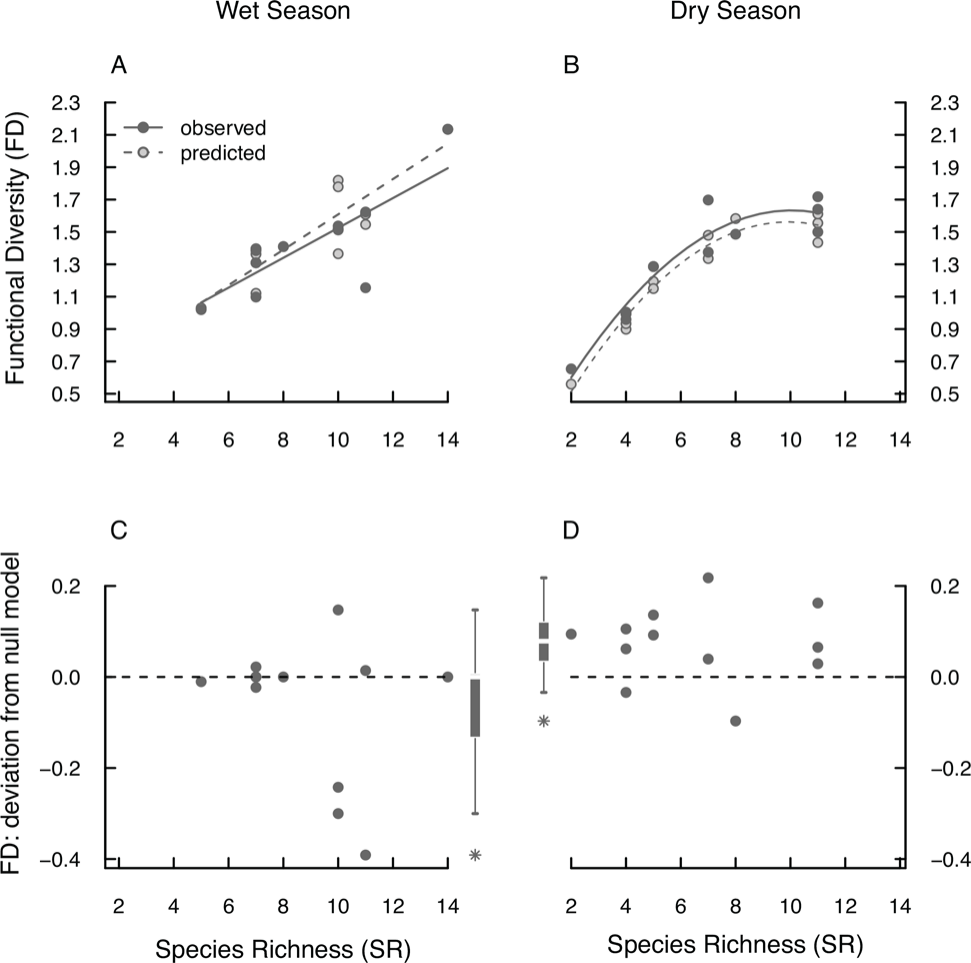
Comparing null models and observed FD from tadpole assemblages from RNP. Given are observed (dark grey filled circles, continuous regression line) and null model FD (light grey circles with dark margins, dashed line) along the observed SR gradient for the wet (A) and the dry season (B). Each symbol represents one tadpole assemblage. (C) and (D) show the differences between null model (dashed line) and observed values of FD (grey circles) of the wet and dry season, respectively. Values above or below the line show observed values being higher (high FD) or lower (low FD) than predicted, respectively. As graphical summary, the respective box-whisker plots are provided next to the scatter plot with outliers indicated as asterisks. Differences were not significant in the wet season; in the dry season assemblages show significantly higher FD than expected.

As observed for SR, FD decreased from the wet to the dry season. However, the observed relative loss of FD (8%) differs from the loss predicted by the null models (17%) based on the observed loss in SR (paired t-test, t = −2.34, df = 11, p = 0.04; Fig 3). This difference suggests that species loss and/or species turnover from the wet to the dry season is non random with respect to the species traits, and it is explained by the structure of the tadpole assemblages of the dry season. Firstly, tadpole assemblages do not show functional redundancy in the wet season, as both observed and null model FD show a linear relationship with SR (Fig 4A; linear regressions; null model: R^2^ = 0.78, F_1,10_ = 35.8, p_model_ < 0.001, p_intercept_ = 0.012, p_SR_ < 0.001; observed: R^2^ = 0.63, F_1,10_ = 17, p_model_ = 0.002, p_intercept_ = 0.015, p_SR_ = 0.002). Also, there is no difference between observed and null model FD (Fig 4C; paired t-test, t = −1.43, df = 11, p = 0.18). In the dry season, however, the tadpole assemblages are characterised by functional redundancy as indicated by curvilinear relationship of FD with SR (Fig 4B; polynomial regressions; null model: R^2^ = 0.96, F_2,9_ = 114, p_model_ < 0.001, p_SR_ < 0.001, p_SR^2_ < 0.001, observed: R^2^ = 0.91, F_2,9_ = 45.8, p_model_ < 0.001, p_SR_ < 0.001, p_SR^2_ < 0.005). Furthermore, these assemblages show high functional diversity, i.e. observed FD values are higher than predicted by the null model (Fig 4D; paired t-test; t = 2.99, df = 11, p = 0.012).

In a nutshell, the loss and/or turnover of species in tadpole assemblages in RNP from the wet to the dry season is non random with respect to species traits with patterns of high FD (compared to the null model) and higher functional redundancy (with increasing SR among sites) in the dry season, whereas in the wet season FD does not provide any more or different information than SR.

### Phylogenetic diversity

**Fig 5:**
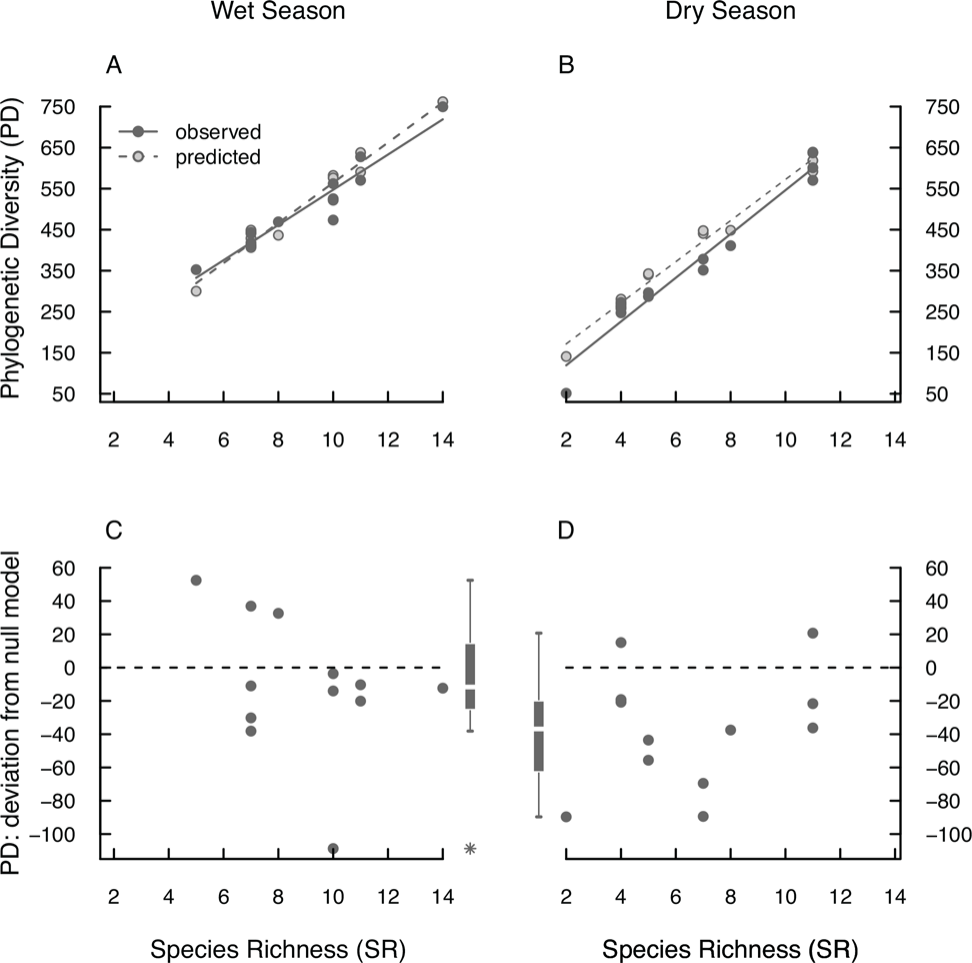
Comparing null models and observed PD from tadpole assemblages from RNP. Given are observed (dark grey filled circles, continuous regression line) and null model PD (light grey circles with dark margins, dashed line) along the observed SR gradient for the wet (A) and the dry season (B). Each symbol represents one tadpole assemblage. (C) and (D) show the differences between null model (dashed line) and the observed values of PD (grey circles) of the wet and dry season, respectively. Values above or below the line show observed values being higher (phylogenetic overdispersion) or lower (phylogenetic clustering) than predicted by null models, respectively. As graphical summary, the respective box-whisker plots are provided next to the scatter plot with outliers indicated as asterisks. Differences were not significant in the wet season; in the dry season, assemblages show significant phylogenetic clustering.

The observed relative loss of PD (28%) of tadpole assemblages was stronger than predicted (23%) by null model assemblages (Fig 3; paired t-test; t = 3.08, df = 11, p = 0.011). To identify the reason for this deviation of the observed data from the null model, we focused on PD in the wet and the dry season separately. In the wet season, both observed and null model PD show a linear relationship with SR indicating no phylogenetic redundancy (Fig 5A; linear regressions; null model: R^2^ = 0.96, F_1,10_ = 267.7, p_model_ < 0.001, p_intercept_ = 0.022, p_SR_ < 0.001; observed: R^2^ = 0.93, F_1,10_ = 123.8, p_model_ < 0.001, p_intercept_ = 0.008, p_SR_ < 0.001). The observed PD of tadpole assemblages in the wet season does not differ from the predicted values (Fig 5C; paired t-test; t = −0.88, df = 11, p = 0.4). Therefore, there is neither phylogenetic clustering nor overdispersion in tadpole assemblages in the wet season. In the dry season, null assemblages predict that PD will be highly related to SR with a trend to curvilinearity (Fig 5B; polynomial regression; R^2^ = 0.99, F_2,9_ = 378.2, p_model_ < 0.001, p_intercept_ = 0.82, p_SR_ < 0.001, p_SR^2_ = 0.06). Indeed, a similar relationship of SR and PD is shown for the observed assemblages. However, as our data set includes highly influential data points to be considered in regression analysis we consider our model conservative and do not refer it to the polynomial model (Fig 5B; simple linear regression; R^2^ = 0.96, F_1,10_ = 227.5, p_model_ < 0.001, p_intercept_ = 0.63, p_SR_ < 0.001). More of relevance, however, is that PD of tadpole assemblages in the dry season is significantly lower than predicted by null models (Fig 5D; paired t-test, t = −0.64, df = 11, p = 0.004). Therefore, in the dry season tadpole assemblages show phylogenetic clustering, i.e., they are assembled by species that are more closely related to each other than expected by chance.

Summarising the results concerning PD, species loss and/or turnover from the wet to the dry season are non-random with respect to the degree of relatedness of the species in an assemblage. This non-randomness is expressed in phylogenetic clustering in the tadpole assemblages in the dry season (Fig 5B and 5D).

There was no spatial autocorrelation of the diversity measures in any of the seasons (all p range from 0.22 to 0.93).

## Discussion

Seasonality as observed in the tropical rainforest of Madagascar causes changes in SR, FD, and PD of tadpole assemblages within stream habitats. All of these measures of diversity were observed to decrease in the dry season compared to the wet season. However, FD and PD do neither change congruently to SR nor to each other. Furthermore, changes as observed differ significantly from predictions by null models. The loss of FD from the wet to the dry season was smaller than predicted, while PD changed more strongly than predicted. Whereas in the wet season all three measures (SR, FD, PD) provide similar information on tadpole assemblages, strong differences are found in the dry season. In the dry season the assemblages are characterised by functional redundancy (among assemblages—with increasing SR), high FD (compared to a null model), and low PD (phylogenetic clustering; compared to a null model). This suggests that both tadpole functional traits and tadpole relatedness are important determinants in the change of assemblage composition following seasonal climatic changes.

### Species richness decreases from wet to dry season

From the warm and wet to the climatically less suitable, cooler dry season [38] species richness in tadpole assemblages at Ranomafana National Park (RNP) decreased significantly. Tropical seasonality has been shown to affect frog activity and accordingly tadpole SR at ponds [36, 38] but not always at streams [35, 62], indicating often more stable conditions in streams than in ponds [63]. In Madagascar, frogs in the dry season are much less active [37] and might also be more susceptible to infection with pathogens such as the amphibian chytrid fungus [64]. Beside the loss of SR, assemblages can change in composition by species turnover [32], as it is the case in the observed RNP tadpole assemblages. This is often due to species that are seasonal specialists [65]. However, no species in this study can be classified as being strictly specialised on one season. We assume that in most RNP stream breeding species, reproduction takes part throughout the year, at least occasionally. Also, some larvae observed in the dry season (i.e., species with long larval development) might derive from clutches deposited in the wet season, blurring possible patterns of specialists reproducing in one of the seasons only.

### Functional diversity decreases less than expected from wet to dry season

As expected from the observed decrease of SR from wet to dry season, tadpole assemblages show a decrease of FD. This decrease of FD is lower than predicted by a null model, and accordingly assemblages are presumed to have a relatively higher FD in the dry season. This study therefore shows that a seasonal loss of species from assemblages is trait-related, as it has also been observed in tropical beetles [66], and mainly such species remain in the assemblages that are functionally different [24]. This is an indication that interspecific competition might play a role in shaping these assemblages ([24] but see discussion of PD below) in the dry season, but less so in the wet season. So far, interspecific competition was assumed to not being important at all in shaping stream tadpole assemblages [25, 63, 67]. These studies, however, were all conducted during the wet season, and agree with the wet season data presented herein. Evidence for competition in tadpole assemblages came so far only from temporary ponds [68–71]. In the dry season in Malagasy rainforest streams reduced food availability, e.g., due to slower algal growth at low temperatures, or lower influx of nutrients, may induce or increase competition among tadpole species despite lower SR in this time, i.e., less potentially competing species.

Additionally, these assemblages show functional redundancy (when comparing FD of assemblages within the dry season): the more species occur in an assemblage, the more they overlap in their ecological functions. We here find this functional redundancy pattern in the dry season assemblages, and it previously has been observed also in the wet season [25]. The lack of redundancy in the wet season assemblages analysed herein might be due to a lower range of SR compared to the previous study [25] which included 29 assemblages of partly much higher SR, illustrating that the probability of detecting redundancy depends on the level of SR [25, 72]. We interpret the missing signal of wet season functional redundancy in the smaller data set analysed herein as an indication of a weaker redundancy effect as compared to the dry season assemblages.

### Phylogenetic diversity decreases stronger than expected from wet to dry season

Phylogenetic diversity of stream tadpole assemblages also decreases with a loss of SR. Contrary to the observed pattern for FD, the loss of PD is stronger than predicted by a null model and assemblages show phylogenetic clustering [29] in the dry season (but not in the wet season). Hence, those species that are less closely related to the remaining species disappear from the assemblages during the shift from the wet to the dry season. Such phylogenetic clustering has also been observed in several bacterial [28], insect [73] and plant assemblages [74, 75].

Following the arguments of the competition-relatedness hypothesis [17, 26] the species within the dry season assemblages are more closely related than expected and therefore they should also be more functionally similar. This would indicate an influence of environmental filtering in species assembly [9, 28]. However, the inverse pattern observed for FD (i.e., lower decrease than expected of FD vs. higher decrease than expected of PD) is contradictory to this hypothesis. High FD in Malagasy stream tadpoles actually indicates that competition plays a more significant role than environmental filtering, and this is not necessarily in conflict with the PD data. In fact, the observed pattern of phylogenetic clustering (low PD) might arise under competition if certain clades are stronger competitors than others irrespective of other traits [27]. Also, the niche conservatism hypothesis [76] might provide an alternative explanation for the phylogenetic clustering in the dry season. Accordingly, the phylogenetically related species occurring during the dry season might have similar ecological traits allowing them to cope with the harsher conditions in the dry season where other species not having these traits are excluded. During evolutionary time, these phylogenetically related species might have conserved their niches more similar to each other than the other distant related species leading to a closely related dry season assembly, i.e. to phylogenetic clustering.

An additional point needs to be considered: life history variables such as larval developmental time and breeding phenology might be phylogenetically conserved traits, and accordingly those clades, e.g., with quick larval development and breeding at the onset of the rainy season would have completed metamorphosis and left the streams in the dry season while the clades with slow larval development would still remain in the streams throughout the dry season. The remaining, accordingly more closely related species then would face high competition. Obtaining more reliable natural and life history data for Madagascan frogs will be paramount to better understand such assembly processes of tadpole communities at specific points in time.

### Opposing patterns of functional and phylogenetic diversity are not necessarily contradictory

The morphological and therefore functional similarity of members of RNP tadpole assemblages does not fully reflect their relatedness. This is evident during the dry season, when assemblages have lower SR, FD, and PD than in the wet season, and tadpoles are more closely related but functionally more different than expected by chance. This opposing pattern of FD and PD is mutually contradictory only at first glance, and several factors might explain why FD, PD and SR are not necessarily interchangeable.

Firstly, FD and PD of assemblages may be partially decoupled from SR, e.g., if a decrease in SR of assemblages is associated with species turnover [32, 77], with species being replaced by others that are less closely related but functionally more similar than the remaining species in the assemblage, and vice versa. Although Madagascan tadpole assemblages do show species turnover and tadpoles may have been replaced by species closely related to remaining species but showing a different morphology, we see a clear link of both FD and PD to SR, and the loss of SR had clearly a stronger effect than the species turnover. In fact, Flynn et al. [10] argues that both FD and PD may be linked to SR, but not directly to each other. However, there are several studied systems with a clear phylogenetic signal in functional traits (e.g., [20, 78] and others cited above). Additionally, if genetic markers are linked to a function (e.g., protein coding genes in microorganisms) PD and FD may be even more strongly correlated to each other than to SR [79]. In Madagascan tadpoles, some closely related groups indeed show a broader range of traits than others which is not necessarily linked to their species richness (e.g., [80–82]). There are several accounts of homoplasy in morpho-functional traits in Madagascan tadpoles [83] although in general, tadpole morphology largely fits phylogeny ([84] and references cited above). As the mentioned exceptions are randomly distributed between the taxonomic groups included in this study, they may not necessarily have influenced the results.

Secondly, a mismatch of FD and PD may appear if some local assemblages comprise species with comparable origin while others comprise species with different origin [15, 16, 85]. The Madagascan anuran fauna originated by five independent colonization events [86] but all species in our study belong to one of these clades, i.e., the Madagascar-Comoroan endemic Mantellidae [40]. The species in the dry and wet season assemblages all underwent comparable evolutionary histories in the eastern rainforest [50], and a bias caused by different origins is therefore unlikely.

Thirdly, it is evident that FD is based on a defined set of ecological traits that may only be a part of the traits covered by phylogeny. The additional traits included in PD, however, may be of relevance, or not, for species assembly. If too many ecologically non-relevant traits are covered by PD they might, as they are included in the analysis as describing factors, mask the information of the relevant traits [21]. Additionally, some functional traits of relevance for species assembly may lack a phylogenetic signal [85, 87] or vary in this signal [20]. Also, PD may include one or a few key traits that simply have not been considered for FD. If so, environmental filtering (or competition) may act and select on these traits and will cause phylogenetic clustering; the remaining species are rather similar regarding these traits (covered by PD but not FD) but also rather different regarding the remaining traits included in FD analysis. If so, a pattern as observed in our study may appear.

Fourthly, Swenson [21] related the applicability of PD to the phylogenetic scale. Which scale is appropriate surely depends on the system studied. In terms of their general morphology and ecology, tadpoles are rather similar to each other (compared to, e.g., plant assemblages with grasses, herbs, and legumes) and adaptive evolution may play a strong role in our study system. However, most tadpoles can easily be assigned to a specific phylogenetic group just by their general appearance indicating an appropriate phylogenetic scale in this study. Additionally, we do see a pattern in PD analysis indicating the relevance of phylogeny. The question of scale is of course also of relevance for FD. This study and data of Strauß et al. [25] do show FD patterns and therefore confirm the scale also appropriate for FD studies.

### Conclusions

The harshness of environmental conditions may influence whether traits cluster (more stressful conditions) or overdisperse (less stressful conditions) [19]. Our results illustrate the necessity of observing the diversity of assemblages at different environmental conditions, i.e., in different seasons. Assemblages without seasonal specialists and persisting at the same site during the year vary strongly in their functional and phylogenetic diversity, and obviously their assembly rules. In general, FD and PD provide more information than SR without being interchangeable. Both measures contribute differently to describe diversity. The appropriate selection of traits for FD on the one hand and the missing selection of traits in PD on the other hand remains a major challenge in studies based on these measures. However, if FD is used to indicate assembly rules, the underlying traits must be of relevance (i.e., for competition and environmental filtering) and allow interpretation, respectively. Beside the mentioned advantages of using PD in diversity studies, however, the interpretation of observed patterns is diverse and should be related to FD. Our data, however, are unambiguous in suggesting that the loss of anuran larvae from the more suitable (i.e., warm-wet) season to the harsher (i.e., cool-dry) season is not random. Although there are no data supporting whether differences in assemblage composition are either based on the duration of larval development or phenological differences in reproductive activity, our results indicate that competition influences the composition of these assemblages at least periodically.
